# Same-sex Pairs Retain Their Reproductive Capacity as a Potential Opportunity for Individual Reproductive Success in Termites

**DOI:** 10.1093/jisesa/ieac073

**Published:** 2023-02-09

**Authors:** Jia Wu, Jinpei Wang, Yonghui Wang, Ali Hassan

**Affiliations:** Applied Research Center for Life Science, Xi’an International University, Xi’an, Shaanxi 710077, China; College of Medicine, Xi’an International University, Xi’an, Shaanxi 710077, China; Applied Research Center for Life Science, Xi’an International University, Xi’an, Shaanxi 710077, China; College of Medicine, Xi’an International University, Xi’an, Shaanxi 710077, China; Engineering Research Center of Personalized Anti-aging Health Product Development and Transformation, Universities of Shaanxi Province, Xi’an, Shaanxi 710077, China; College of Engineering, Xi’an International University, Xi’an, Shaanxi 710077, China; Hubei Insect Resources Utilization and Sustainable Pest Management Key Laboratory, Huazhong Agricultural University, Wuhan 430070, Hubei, China

**Keywords:** homosexuality, colony fusion, life span, gonadal development, termite

## Abstract

In eusocial termites, successful pairing is an essential element of dispersal and distribution after the departure of alates from natal colonies. Two situations could arise during the pairing process: mixed-sex pairs and same-sex pairs. However, most previous studies focused on mixed-sex pairs, overlooking groups formed by same-sex pairings, especially potential fecundity (the total number of oocytes or ovarioles), oogenesis and the development stage of oocytes of females in female–female pairs, and spermatogenesis and testis development of males in male–male pairs. In this study, through experimentation, we investigated the reproductive ability of virgin dealates based on various pairing types as mentioned above. We found that the life spans of virgin dealates can cover 1 yr or even more when they establish a nest with a partner, which is more than 10-fold longer than the life span of individuals establishing a colony alone. After 1 yr of pairing, the potential fecundity of virgin same sex dealates did not degenerate significantly compared with newly emerged dealates, including the number of ovarioles, size of testis, oogenesis, and the development stage of the oocytes. Moreover, when individuals of same-sex pairings experimentally changed into mixed-sex pairs after 1 yr, the eggs produced in the colony hatched into larvae. These findings suggest that dealates which through same-sex pairs retain fecundity after 1 yr have more reproductive potential than dealates that failed to pair with heterosexuals, shedding light on the ecological significance of homosexual behaviors in terms of the successful extension and fecundity of eusocial termites.

## Graphical Abstract

**Graphical Abstract:**
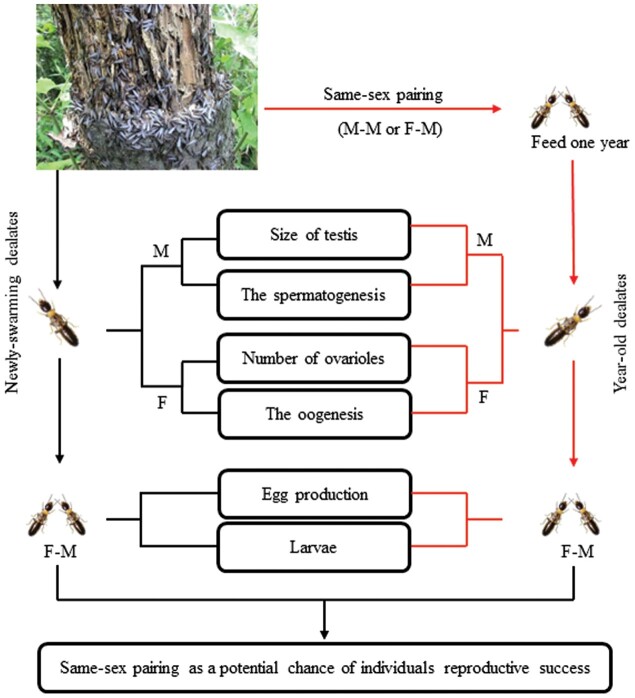

A new termite colony is usually established by one female dealate (primary queen) and one male dealate (primary king) during swarming, which then gives rise to other colony members (Thorne et al. 1999, Korb and Hartfelder 2008). However, recent studies suggested that homosexual behaviors in termites is common during courtship, copulation, and pairing (Li et al. 2015, Mizumoto et al. 2016). The presence of this behavior is a waste of reproductive resources for monogamous termites—it cannot produce offspring. The significance and existence of potential reproductive behavior of same-sex pairings during reproduction are still poorly understood.

Same-sex pairing behaviors have been attributed to errors in sexual recognition (Burgevin et al. 2013, Scharf and Martin 2013), but this is unable to explain same-sex pairing in termites (Mizumoto et al. 2016, 2022). Mixed-sex pairs run in tandem to find a suitable nest site, and the male will follow the female when they encounter each other. Tandem running is synchronized; a pair moves as one. When a couple accidentally separates, the female pauses to wait for the male while the male continues to move to look for the female (Vargo and Husseneder 2009, Mizumoto and Dobata 2019). However, same-sex pairs, especially male–male pairs, run in tandem to compete for a beneficial position. Two individuals of same-sex pairs in tandem often try to follow each other to form circles, because the individual at the back of a male–male tandem is likely to win a female if and when encountering one (Li et al. 2015). This behavior may even result in a switch of leader–follower positions (Mizumoto et al. 2022).

Although recent studies have suggested that same-sex behavior results in a dilution of predation risk because predators such as ants do not attack two dealates (Mizumoto et al. 2016, Mizumoto and Dobata 2019), this cannot explain the establishment of a same-sex colony after a tandem (Mizumoto et al. 2016). Same-sex nest building in females having the ability of parthenogenesis was considered to produce offspring by this route (Matsuura et al. 2004). In fact, however, parthenogenesis has been reported for only about eleven termite species in four families (Ishitani and Maekawa 2010, Matsuura 2011, Yashiro and Matsuura 2014, Fougeyrollas et al. 2015, Vargo 2019). For most termites, it does not make sense for females to build nests with females, because the eggs produced by females paired with females do not hatch, so the females have no fitness (Kawatsu and Matsuura 2012). Males building nests cannot reproduce.

Most termite species exhibit a biased sex ratio (Kobayashi et al. 2013, Li et al. 2014). Given the uniqueness of monogamy, extremely high predator risks and other factors, most individuals are not able to found a new colony with an opposite sex partner even if pairing conditions are optimal (Wu et al. 2020). Thus, rejecting a homosexual partner and overzealously seeking an opposite sex partner may result in the loss of all fitness as a colony that is established by a single individual is mortal (Liu et al. 2019). In contrast, accepting a homosexual partner permissively may lead to survival opportunities and even reproduction opportunities through colony fusion. Colony fusion is relatively common in *Reticulitermes* termites: field studies have estimated that fusion occurs in 2.3–31% of mature colonies of *Reticulitermes flavipes* Oshima (DeHeer and Vargo 2004, 2008; Perdereau et al. 2010; Mizumoto et al. 2016). There are complex assemblages of microorganisms (bacterial and fungal pathogens) in termite nests. The individuals are incapable of grooming themselves aside from their antennae, and must rely on nest mates. Thus, the single individuals are unable to survive without mates, so colony fusion is not possible for a sole termite. On the contrary, the life span of individuals is lengthened by same-sex pairs and then colony fusion is possible. Despite these suggestive findings, only a few studies have experimentally tested the fecundity of same-sex pairs. Meanwhile, it is not yet clear how well the gonads develop and whether same-sex pairs can reproduce when encountering individuals of the opposite sex, without the establishment of a colony of offspring.

In the present study, the life span of each individual forming same-sex pairs was measured in Reticulitermes *flavipes* Oshima. Gonadal developments were evaluated by an index including the number of ovarioles, size of testis, oogenesis, and spermatogenesis. Reproductive ability was evaluated by experimental pairing treatments. Our results contribute to an explanation of the adaptive significance that ensures the persistence of the abnormal reproductive behavior of same-sex pairing.

## Materials and Methods

### Termites

*R. flaviceps* colonies used in this study were collected from Shizi hill, Wuhan city, Hubei province, China during the swarming seasons (March to April) in 2019 and 2020. The parts of the nest with alates were brought to the laboratory, and dispersal flight was initiated using methods described previously (Wu et al. 2020). Female and male dealates were divided via the shape of the seventh abdominal sternite (Miyata et al. 2004, Wu et al. 2013), and individuals of same sex were placed in one Petri-dish (Φ= 12 cm) until the experiments were commenced.

### Life spans of Virgin Dealates

The reproductive success of a virgin dealate is directly influenced by the life spans of individuals and partners, because single individuals do not survive by themselves. Even if a pair is formed, the survival of a dealate depends on the survival of another dealate and almost all surviving dealates cohabit with another until a worker is differentiated in the colony (Mizumoto et al. 2016). The life spans of initial colonies that were established by two virgin dealates (two males or two females) were observed in order to assess the possibility of encounters between the dealates that previously swarmed and recently swarmed. We set up 900 pairs of dealates including female–female, male–male, and female–male pairs derived from nine colonies. Each pair was placed into a 120 ml transparent cylindrical vial (φ = 3 cm) with moistened filter paper and pine wood at 20–26°C in constant darkness. We recorded colony survival during each month for 1 yr. A colony was defined as surviving if two or more live individuals were present. Single individuals did not survive by themselves. Survival of an individual in a same-sex pair depended on the survival of another individual and almost all surviving individuals cohabited with another one. We used generalized linear models (GLM) to investigate survival rates of pairings. In these models, explanatory variable (survive rate) was treated as a fixed factor and colony as a random factor.

### Observations of Gonadal Development

To evaluate the possible fecundity of individuals, two types of dealate individuals were studied: one was swarmed from the natal colony (newly swarming dealates [ND]), and the other was fed and then kept as a pair for a year (year-old dealates [YD]). Their ovarian development was evaluated by counting the numbers of ovarioles, and their testis development was evaluated by measuring its diameter. The two types of individuals (ND and YD) were used in the observations of gonadal development. We undertook 20 replicates for each group in the observations of ovarian development and 18 replicates for each group in the observations of testis development. The entire ovary and testis were stripped from the abdomen of dealates under digital microscopes. We recorded the number of ovarioles and the size of testis. A *t*-test was applied to analyze the difference in the number of ovarioles and size of testis between ND and YD.

The oogenesis and spermatogenesis of the ND and YD was evaluated with hematoxylin-eosin (HE) staining. The fixed samples were dehydrated in an ascending ethanol series and embedded in paraffin. Longitudinal 7-μm sections were processed by a microtome and collected on polylysine-coated slides. The deparaffinized and rehydrated sections were stained with HE. The sections were observed using digital microscopes (Olympus Corporation, Japan), and the number of vitellogenic oocytes was counted to evaluate the level of ovarian maturation. The number of spermatozoa was counted to evaluate the level of testis maturation.

### Experimental Pairing Treatments

To evaluate the reproductive ability of ND and YD, we established two colony types: one was ND of female–male dealate pairs and the other was YD of female–male dealate pairs. We prepared 30 replicates for each type of pairing, with each pairing being reared in a 120 ml transparent cylindrical vial (φ = 3 cm) with moistened pine sawdust at 20–26°C in constant darkness. Water was supplied when required. We performed observations every 2 d, and the number of eggs and larvae were recorded. We used a GLM to analyze differences. In the model, the eggs and larvae were treated as a fixed factor and colony as a random factor.

## Results

**Fig. 1.**
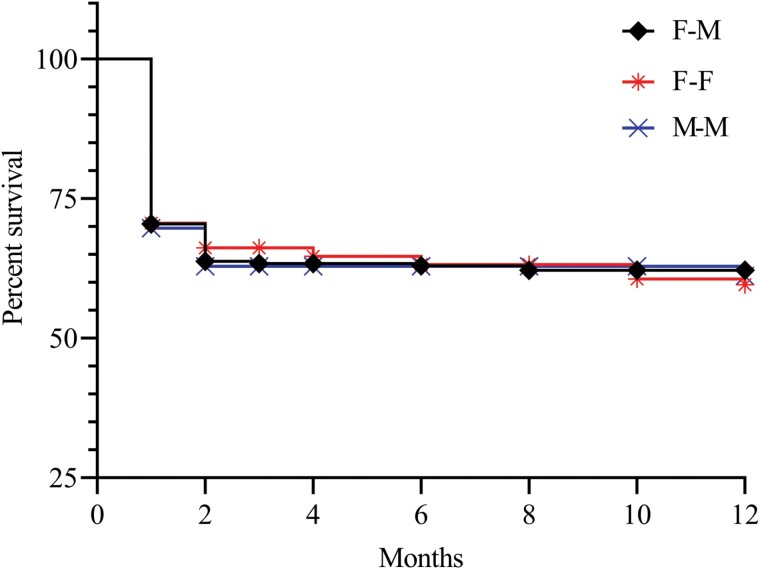
The survival rate of homosexual pairs and heterosexual pairs. Survival was no significantly different in female–female, male–male, and female–male colonies (F = 0.13, *P* = 0.87).

In terms of the formation of same-sex pairs (including female–female and male–male pairs), virgin dealates of *R. flaviceps* did not die immediately when they failed to mate in the swarming season. In this study, three types of pairing (male–male, female–female, and female–male) had the same-trend of survival curves with a greater than 30% death rate in the first month and relatively stable start from the second month. Within 1 yr, the survival rates of the female–female, male–male, and female–male pairs were not significantly different, and more than 50% of pairings survived for at least 1 yr (Fig. 1, *F* = 0.13, *P* = 0.87). There was no significant difference in pairing survival rates among different colonies (*F* = 0.17, *P* = 0.68). This indicates that two virgin dealates (female–female and male–male) require only a suitable patch to establish a nest and survive 1 yr or longer.

**Fig. 2.**
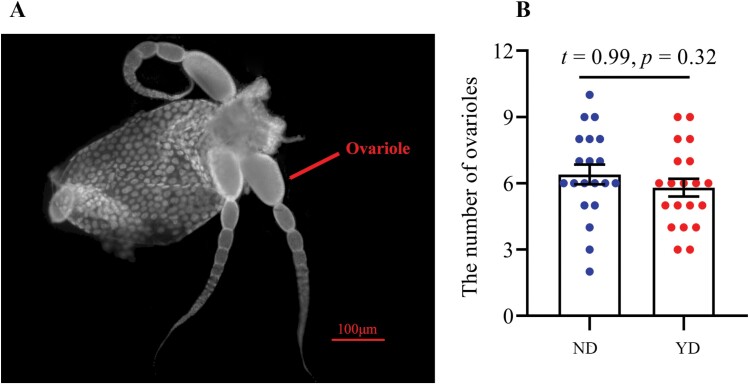
The morphological characteristics and number of ovarioles in the ND and YD. (A) the morphological characteristics of ovarioles; (B) the number of ovarioles the ND and YD (*n* = 20). The number of ovarioles in the ND and YD was no significant difference (Fig. 2B; *t* = 0.99, *P* = 0.32). ND, newly dealate; YD, year-old dealate.

**Fig. 3.**
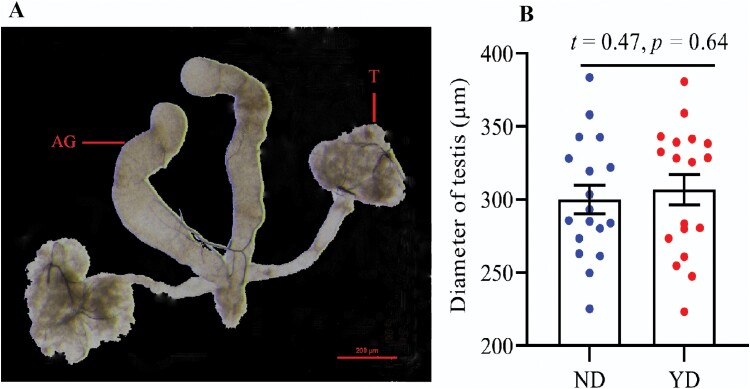
The morphological characteristics and size of testis in ND and YD. (A) The morphological characteristics of testis; (B) the diameter of testis in ND and YD (*n* = 18). The diameter of testis in ND and YD was no significant difference (Fig. 3B; *t* = 0.47, *P* = 0.64). T, testis; AG, accessory gland; ND, newly dealate; YD, year-old dealate.

Compared with ND individuals, the function of the ovary and testis in YD individuals did not degenerate significantly. The mature eggs with shell were found at the end of ovarioles in ND and YD individuals (Fig. 2A). There was no significant difference in the number of ovarioles between ND and YD (Fig. 2B; *t* = 0.99, *P* = 0.32). This result showed that virgin females are able to lay mature eggs after 1 yr of post-swarming. For males, there was no significant difference in the diameter of testis between ND and YD (Fig. 3B; *t* = 0.47, *P* = 0.64).

**Fig. 4.**
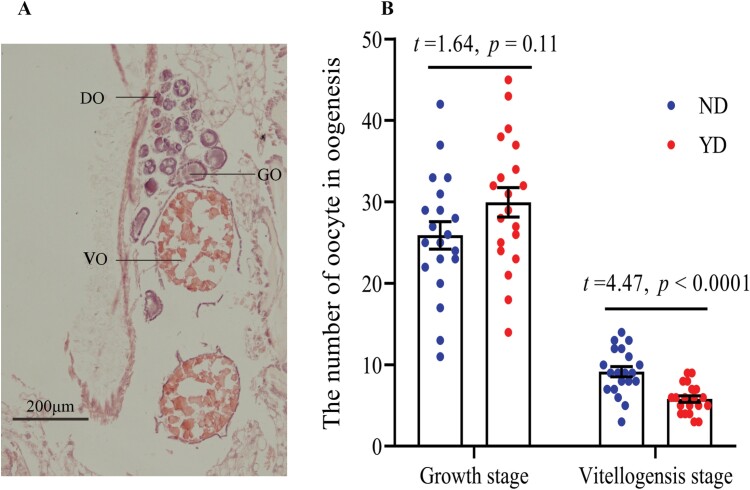
The oogenesis and the number of oocytes in different stage. (A) the oogenesis; (B) the number of oocytes in oocyte growth stage and oocyte vitellogenesis stage. DO, oogonium differentiation stage; GO, oocyte growth stage; VO, oocyte vitellogenesis stage.

**Fig. 5.**
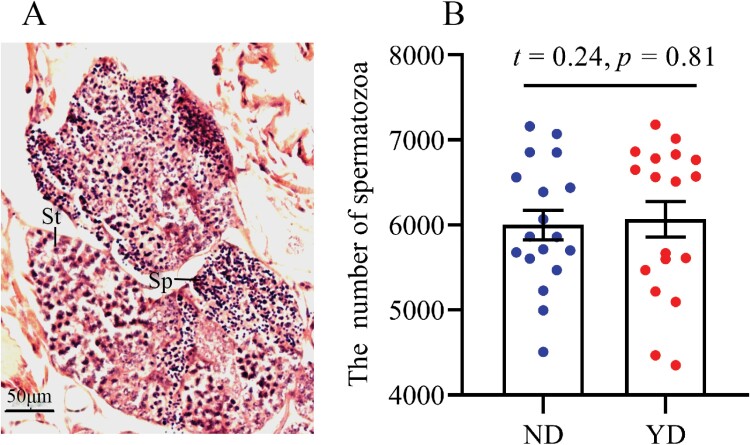
The spermatogenesis and the number of spermatozoa in testis. (A) The spermatogenesis; (B) the number of spermatozoa in testis. St, spermatids; Sp, spermatozoa A: Egg production in different pairs; B: the number of larvae in different pair combinations. Newly dealates pairs laid more egg (*F* = 23.99, *P* < 0.001) and larvae (*F* = 12.58, *P* = 0.002) than year-old dealates pairs. ND, newly dealate; YD, year-old dealate.

Three stages of oogenesis including the oogonium differentiation stage (DO), oocyte growth stage (GO), and oocyte vitellogenesis stage (VO) were observed in the ovaries of ND and YD individuals at the same time (Fig. 4A). The oocytes in the oogonium differentiation stage were tightly packed and unclearly defined. At the oocyte growth stage, each oocyte was surrounded by a layer of thick follicular cells (Fig. 4A). The oocyte of the growth stage was observed more in the ovaries of YD than ND, but we could not detect a difference in the number of oocytes in the growth stage between ND and YD (Fig. 4B; *t* = 1.64, *P* = 0.11). Oocyte development reached the vitellogenesis stage when the size of the oocytes was largest, and each oocyte was surrounded by a thin follicle cell layer indicating that the oocytes had matured (Fig. 4A). The vitellogenic oocytes were observed in the ovaries of ND and YD; the number of vitellogenic oocytes was significantly lower in the ovaries of YD than the ovaries of ND (Fig. 4B; *t* = 4.47, *P* < 0.001). Thin sections through the testes showed different stages of spermatogenesis; spermatids and spermatozoa were observed both in ND and YD (Fig. 5A). There was no significant difference in the number of spermatozoa between ND and YD (Fig. 5B; *t* = 0.24, *P* = 0.81).

**Fig. 6.**
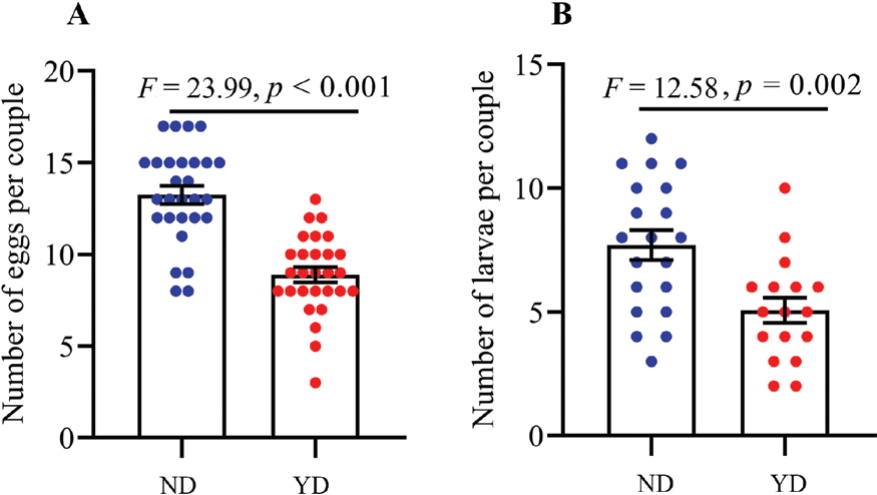
(A) Egg production in different pairs; (B) the number of larvae in different pair combinations. Newly dealates pairs laid more egg (*F* = 23.99, *P* < 0.001) and larvae (*F* = 12.58, *P* = 0.002) than year-old dealates pairs. ND, newly dealate; YD, year-old dealate.

Both pairing colonies laid eggs, but the number of eggs laid per colony by YD were lower than by ND (Fig. 6A; *F* = 23.99, *P* < 0.001). The eggs in the two types of colony can also hatch into larvae and there was no significant difference in hatch rate (*F* = 0.24, *P* = 0.87). The number of larvae was significantly lower in the colony established by YD than by ND (Fig. 6B; *F* = 12.58, *P* = 0.002). As a result, eggs and larvae can be produced in colonies established by YD individuals, demonstrating that in the case of an encounter with a heterosexual partner, the virgin individuals can also mate and produce living offspring 1 yr post-swarming.

## Discussion

Our results showed that in *R. flaviceps*, dealates can survive longer and retain their reproductive capacity through same-sex pairings (male–male and female–female). In termites, searching for a partner and establishing nests during swarming is necessary for survival. Considering the reproductive processes of termites, the searching time for partners in order to mate is closely linked to colony establishment, and is therefore considered a major factor influencing reproductive success (Thomas et al. 2006, Zhang et al. 2021). In the case of a lack of opposite sex individuals, in some termite species two males or two females could encounter and establish a same-sex pairing colony and reproduce through parthenogenesis (Matsuura et al. 2002b, Vargo 2019), fusing the colony later (Deheer and Vargo 2004, 2008; Mizumoto et al. 2016). The life span of paired virgin dealates determines the possibility of colony fusion, because two main factors of fusion are the next time that alates are swarming and that foragers from other colonies are present (Lee et al. 2019). Same-sex pairings could significantly increase the survival of single virgin dealates (Mizumoto et al. 2016). These findings provide a solid foundation for explaining reproduction in 1-yr-old, same-sex pairs of virgin dealates in nature, which mate with newly swarming dealates through colony fusion.

Gonadal development in termites would appear to be the most significant biological process that reflects reproductive differences (Su et al. 2015). Dealates, having activated gonads, are capable of producing offspring, while dealates having inactive gonads are unable to produce offspring. Newly swarming dealates and year-old dealates have similar oogenesis and functions of the ovary. The ovary of virgin dealates continues developing after dealates lay their first brood of mature eggs. Similar patterns of ovary development are also reported in the termite species *R. speratus* (Ishitani and Maekawa 2010). More importantly, the colonies established by 1-yr-old virgin dealates can also produce eggs and living offspring. The differences between YD and ND in this study, including the number of vitellogenic oocytes, laid eggs, and offspring, are possibly caused by the differences in their ovary cycles of development. The reproductive cycle (ovary cycle of development) of primary reproduction is 5–6 mo in an incipient colony, whereby egg laying stops about 1.5–2.5 mo after colony foundation and then resumes sometime prior to 7.5 mo after colony foundation (Ishitani and Maekawa 2010). These results suggest that when the dealates encounter a same-sex individual and establish a nest, they gain an opportunity for reproduction if colony fusion with dealates from the subsequent swarming takes place.

Same-sex pair behavior in a monogamous mating system should incur considerable costs for reproduction (Maklakov and Bonduriansky 2009, Maklakov and Arnqvist 2009). Why, then, are there still same-sex pairs of termites in nature? For termites, reproductive behaviors entail extremely high risks, especially in searching for a mate (Matsuura et al. 2002a, Raina et al. 2003, Wu et al. 2020). Risk of predation for single individuals was twice that of tandem dealates. Reducing the risk of predation and increasing survival rates is an important reason why same-sex pairs naturally occur (Li et al. 2015, Mizumoto et al. 2016). The other reason is exemplified in *Reticulitermes*, which lives and nests in rotting wood where there are complex assemblages of microorganisms (bacterial and fungal pathogens) (Rosengaus et al. 2003, Matsuura 2011). An individual is incapable of grooming themselves aside from their antennae, and must rely on nest mates (Liu et al. 2019). Thus, dealate individuals are unable to survive on their own, but those that make nests with another homosexual partner survive much longer. Pairing with another homosexual partner is not the best option, but it gives mate-less termites a chance to survive in the hope of finding a mate.

Dealates have long life spans after they establish a nest with a same-sex partner. We therefore speculate that long life may provide a foundation for colony fusion between same-sex pairs and foraging workers. A mature subterranean termite colony may contain thousands of workers, and the foraging territory ranges from dozens to thousands of centiares (Su and Scheffrahn 1988, Nobre et al. 2007). It is highly likely that foraging workers integrate with a same-sex pair colony if they encounter one. Any worker in a colony has the potential to develop secondary reproduction (Lainé and Wright 2007, Korb and Hartfelder 2008, Hartke and Baer 2011). A dealate, as a colony founder, provides the primary reproduction for the colony. Both secondary reproduction and primary reproduction is undertaken by sexually mature individuals, which are likely to mate under certain conditions (Korb and Hartfelder 2008, Hartke and Baer 2011). Thus, we speculate that this may be a new mode of colony foundation for termites except for colonies established by a primary king and primary queen.

Same-sex pairs have long life spans providing the possibility for hybridization with a heterospecific partner. The differentiation of swarming is an important mechanism of reproductive isolation between two termite species (Wu et al. 2020). Our results showed that same-sex pairs (male–male and female–female) have high survival rates and survive for at least 1 yr, which could cover all dispersal flight seasons of closely related species. However, some closely related species (e.g., *R. chinensis* and *R. flaviceps*) have similar reproductive behaviors and a lack of preferences for conspecifics when individuals from different species encounter each other (Wu et al. 2020). Thus, the reproductive individuals of the two species may hybridize by establishing a colony through same-sex pairing. However, it is unclear whether reproductive isolation is ineffective and whether hybrid offspring will produce their own offspring in the natural environment. Further studies on the occurrence of natural hybrids are required.

## Conclusions

Our study showed that the life spans of virgin dealates can extend to 1 yr or even more. Potential fecundity of 1-yr-old virgin dealates did not degenerate significantly compared with newly emerged dealates in terms of the number of ovarioles, size of testis, oogenesis, and the development stage of oocytes. The eggs produced in a colony established by two 1-yr-old virgin dealates can hatch into larvae. These findings suggest that there are reproduction opportunities for dealates that fail to mate, an observation that can provide new insights into the reproductive significance of homosexual behaviors in eusocial termites.

## References

[CIT0001] Burgevin, L., U. Friberg, and A. A. Maklakov. 2013. Intersexual correlation for same-sex sexual behaviour in an insect. Anim. Behav. 85: 759–762.

[CIT0002] Deheer, C. J., and E. L. Vargo. 2004. Colony genetic organization and colony fusion in the termite *Reticulitermes flavipes* as revealed by foraging patterns over time and space. Mol. Ecol. 13: 431–441. 14717897 10.1046/j.1365-294x.2003.2065.x

[CIT0003] Deheer, C. J., and E. L. Vargo. 2008. Strong mitochondrial DNA similarity but low relatedness at microsatellite loci among families within fused colonies of the termite *Reticulitermes flavipes*. Insect Soc. 55: 190–199.

[CIT0004] Fougeyrollas, R., K. Dolejsova, D. Sillam-Dusses, V. Roy, C. Poteaux, R. Hanus, and Y. Roisin. 2015. Asexual queen succession in the higher termite *Embiratermes neotenicus*. Proc. Biol. Sci. 282: 20150260. 26019158 10.1098/rspb.2015.0260 PMC4590441

[CIT0005] Hartke, T. R., and B. Baer. 2011. The mating biology of termites: a comparative review. Anim. Behav. 82: 927–936.

[CIT0006] Ishitani, K., and K. Maekawa. 2010. Ovarian development of female-female pairs in the termite, *Reticulitermes speratus*. J. Insect Sci. 10: 1194–1112. 10.1673/031.010.19401 PMC3029259 21271845

[CIT0007] Kawatsu, K., and K. Matsuura. 2012. Preadaptation for parthenogenetic colony foundation in subterranean termites Reticulitermes spp. (Isoptera: Rhinotermitidae). J. Ethol. 31: 123–128.

[CIT0008] Kobayashi, K., E. Hasegawa, Y. Yamamoto, K. Kawatsu, E. L. Vargo, J. Yoshimura, and K. Matsuura. 2013. Sex ratio biases in termites provide evidence for kin selection. Nat. Commun. 4: 2048. 23807025 10.1038/ncomms3048

[CIT0009] Korb, J., and K. Hartfelder. 2008. Life history and development--a framework for understanding developmental plasticity in lower termites. Biol. Rev. Camb. Philos. Soc. 83: 295–313. 18979593 10.1111/j.1469-185x.2008.00044.x

[CIT0010] Lainé, L. V., and D. J. Wright. 2007. The life cycle of Reticulitermes spp. (Isoptera: Rhinotermitidae): what do we know? Bull. Entomol. Res. 93: 267–378. 10.1079/ber2003238 12908912

[CIT0011] Lee, S. B., A. Mullins, D. Aguilera-Olivares, T. Chouvenc, and N-Y. Su. 2019. Fused solonies of the formosan subterranean termite (Blattodea: Rhinotermitidae) for laboratory experiments. J. Econ. Entomol. 112: 2311–2315. doi:10.1093/jee/toz154 31165146

[CIT0012] Li, G. H., C. L. Lei, Z. H. Wang, and Q. Y. Huang. 2014. Dynamics of sex ratio, fresh weight and nutrient contents at five developmental stages of alates in the subterranean termite *Reticulitermes chinensis*. Insect. Soc 62: 51–57.

[CIT0013] Li, G. H., L. Liu, C. L. Lei, and Q. Y. Huang. 2015. A trade-off between antipredatory behavior and pairing competition produced by male-male tandem running in three Reticulitermes species. Insect Sci. 22: 560–568. 24963824 10.1111/1744-7917.12150

[CIT0014] Liu, L., X. Y. Zhao, Q. B. Tang, C. L. Lei, and Q. Y. Huang. 2019. The mechanisms of social immunity against fungal infections in Eusocial insects. Toxins (Basel) 11: 244. 31035652 10.3390/toxins11050244 PMC6563085

[CIT0015] Maklakov, A. A., and G. Arnqvist. 2009. Testing for direct and indirect effects of mate choice by manipulating female choosiness. Curr. Biol. 19: 1903–1906. 19853448 10.1016/j.cub.2009.08.058

[CIT0016] Maklakov, A. A., and R. Bonduriansky. 2009. Sex differences in survival costs of homosexual and heterosexual interactions: evidence from a fly and a beetle. Anim. Behav. 77: 1375–1379.

[CIT0017] Matsuura, K. 2011. Sexual and asexual reproduction in termites, pp. 255–278. *In* D. E. Bignell, Y. Roisin, and N. Lo (eds.), Biology of termites: a modern synthesis, vol. 1, Springer Dordrecht, Heidelberg, London, New York.

[CIT0018] Matsuura, K., E. Kuno, and T. Nishida. 2002a. Homosexual tandem running as selfish herd in *Reticulitermes speratus*: novel antipredatory behavior in termites. J. Theor. Biol. 214: 63–70. 11786032 10.1006/jtbi.2001.2447

[CIT0019] Matsuura, K., M. Fujimoto, K. Goka, and T. Nishida. 2002b. Cooperative colony foundation by termite female pairs: altruism for survivorship in incipient colonies. Anim. Behav. 64: 167–173.

[CIT0020] Matsuura, K., M. Fujimoto, and K. Goka. 2004. Sexual and asexual colony foundation and the mechanism of facultative parthenogenesis in the termite *Reticulitermes speratus* (Isoptera, Rhinotermitidae). Insect Soc. 51: 325–332.

[CIT0021] Miyata, H., H. Furuichi, and O. Kitade. 2004. Patterns of neotenic differentiation in a subterranean termite, *Reticulitermes speratus* (Isoptera: Rhinotermitidae). Entomolog. Sci 7: 309–314.

[CIT0022] Mizumoto, N., and S. Dobata. 2019. Adaptive switch to sexually dimorphic movements by partner-seeking termites. Sci. Adv. 5: eaau6108. 31223644 10.1126/sciadv.aau6108 PMC6584256

[CIT0023] Mizumoto, N., T. Yashiro, and K. Matsuura. 2016. Male same-sex pairing as an adaptive strategy for future reproduction in termites. Anim. Behav. 119: 179–187.

[CIT0024] Mizumoto, N., T. Bourguignon, and N. W. Bailey. 2022. Ancestral sex-role plasticity facilitates the evolution of same-sex sexual behaviour. bioRxiv: 1–14. doi:10.1101/2022.06.20.496918 PMC9674213 36346843

[CIT0025] Nobre, T., L. Nunes, and D. E. Bignell. 2007. Tunnel geometry of the subterranean termite *Reticulitermes grassei* (Isoptera: Rhinotermitidae) in response to sand bulk density and the presence of food. Insect Sci. 14: 511–518.

[CIT0026] Perdereau, E., A. -G. Bagne`res, F. Dedeine, and S. Dupont. 2010. High occurrence_of colony fusion in a European population of the American termite *Reticulitermes flavipes*. Insect Soc 57: 393–402.

[CIT0027] Raina, A. K., J. M. Bland, J. C. Dickens, Y. I. Park, and B. Hollister. 2003. Premating behavior of dealates of the formosan subterranean termite and evidence for the presence of a contact sex pheromone. J. Insect Behav. 16: 233–245.

[CIT0028] Rosengaus, R. B., J. E. Moustakas, D. V. Calleri, and J. F. A. Traniello. 2003. Nesting ecology and cuticular microbial loads in dampwood (*Zootermopsis angusticollis*) and drywood termites (*Incisitermes minor, I. schwarzi, Cryptotermes cavifrons*). J. Insect Sci. 3: 31–35. 15841247 10.1093/jis/3.1.31 PMC524670

[CIT0029] Scharf, I., and O. Y. Martin. 2013. Same-sex sexual behavior in insects and arachnids: prevalence, causes, and consequences. Behav. Ecol. Sociobiol. 11: 1719–1730.

[CIT0030] Su, N. Y., and R. H. Scheffrahn. 1988. Foraging population and territory of the Formosan subterranean termite (Isoptera: Rhinotermitidae) in an urban environment. Sociobiology 14: 353–359.

[CIT0031] Su, X. H., J. L. Chen, X. J. Zhang, W. Xue, H. Liu, and L. X. Xing. 2015. Testicular development and modes of apoptosis during spermatogenesis in various castes of the termite *Reticulitermes labralis* (Isoptera:Rhinotermitidae). Arthropod Struct. Dev. 44: 630–638. 26344723 10.1016/j.asd.2015.08.009

[CIT0032] Thomas, G., X. Shelton, H. Ping, A. G. Appel, and T. L. Wagner. 2006. Flight speed of tethered *Reticulitermes flavipes* (Kollar)(Isoptera: Rhinotermitidae) alates. J. Insect Behav. 19: 115–128.

[CIT0033] Thorne, B. L., J. F. A. Traniello, E. S. Adams, and M. Bulmer. 1999. Reproductive dynamics and colony structure of subterranean termites of the genus *Reticulitermes* (Isoptera Rhinotermitidae): a review of the evidence from behavioral, ecological, and genetic studies. Ethol. Ecol. Evol. 11: 149–169.

[CIT0034] Vargo, E. L. 2019. Diversity of termite breeding systems. Insects 10: 52. 30759735 10.3390/insects10020052 PMC6409762

[CIT0035] Vargo, E. L., and C. Husseneder. 2009. Biology of subterranean termites: insights from molecular studies of Reticulitermes and Coptotermes. Annu. Rev. Entomol. 54: 379–403. 18793101 10.1146/annurev.ento.54.110807.090443

[CIT0036] Wu, J., X. Su, X. Kong, M. Liu, and L. Xing. 2013. Multiple male and female reproductive strategies and the presence of a polyandric mating system in the termite *Reticulitermes labralis* (Isoptera: Rhinotermitidae). Sociobiology 60: 459–465.

[CIT0037] Wu, J., H. Xu, A. Hassan, and Q. Huang. 2020. Interspecific hybridization between the two sympatric termite Reticulitermes species under laboratory conditions. Insects 11: 14. 10.3390/insects11010014 PMC7022586 31877914

[CIT0038] Yashiro, T., and K. Matsuura. 2014. Termite queens close the sperm gates of eggs to switch from sexual to asexual reproduction. Proc. Natl. Acad. Sci. USA 111: 17212–17217. 25404335 10.1073/pnas.1412481111 PMC4260566

[CIT0039] Zhang, Z. Y., J. Ren, F. Chu, J. X. Guan, G. Y. Yang, Y. T. Liu, X. Y. Zhang, S. Q. Ge, and Q. Y. Huang. 2021. Biochemical, molecular, and morphological variations of flight muscles before and after dispersal flight in a eusocial termite, *Reticulitermes chinensis*. Insect Sci. 28: 77–92. 32039551 10.1111/1744-7917.12763

